# EXTENDED WORKING LIFE POLICIES: INTERNATIONAL GENDER AND HEALTH PERSPECTIVES, EMPIRICAL AND POLICY LANDSCAPE

**DOI:** 10.1093/geroni/igz038.3026

**Published:** 2019-11-08

**Authors:** Martina Rasticova, Jim Ogg

**Affiliations:** 1 Mendel University, Brno. Czech Republic, Brno, Czech Republic; 2 French National Pension Fund (Caisse nationale d’assurance vieilliesse), Paris, Ile-de-France, France

## Abstract

As populations age, extending the working life appears to be widely accepted and promoted by governments (OECD 2006; 2017). Without exception, all countries with modern economies have responded in one way or another to the financial challenges arising from increased life expectancy and ageing populations. Policies to extend working life are ubiquitous, each based on the premise that unsustainable pension systems must be reformed, and public spending reduced. Although there are diverse perspectives on extended working life, gender and health consistently prevail as key dimensions. To date, policies extending working life have not taken sufficiently into account these two dimensions. A clear example in the case of gender concerns the shift towards equality in retirement ages between men and women. In this presentation, we set the stage by presenting the empirical and policy landscapes across 34 countries that characterise the trend of extended working life from gender and health perspectives.

